# Host selection and forage ratio in West Nile virus–transmitting *Culex* mosquitoes: Challenges and knowledge gaps

**DOI:** 10.1371/journal.pntd.0010819

**Published:** 2022-10-27

**Authors:** Nicola Riccetti, Augusto Fasano, Federico Ferraccioli, Jaime Gomez-Ramirez, Nikolaos I. Stilianakis

**Affiliations:** 1 European Commission, Joint Research Centre (JRC), Ispra (VA), Italy; 2 Department of Biometry and Epidemiology, University of Erlangen-Nuremberg, Erlangen, Germany; Oregon Health and Science University, UNITED STATES

## Abstract

**Background:**

To date, no specific therapy or vaccination is available for West Nile virus (WNV) infections in humans; preventive strategies represent the only possibility to control transmission. To focus these strategies, detailed knowledge of the virus dynamics is of paramount importance. However, several aspects of WNV transmission are still unclear, especially regarding the role of potential vertebrate host species.

Whereas mosquitoes’ intrinsic characteristics cause them to favour certain hosts (host preference), absolute selection is impossible in natural settings. Conversely, the selection carried out among available hosts and influenced from hosts’ availability and other ecological/environmental factors is defined as host selection.

**Methodology/Principal findings:**

In July 2022, we searched PubMed database for original articles exploring host selection among WNV-transmitting *Culex* mosquitoes, the main WNV vector. We considered only original field studies estimating and reporting forage ratio. This index results from the ratio between the proportion of blood meals taken by mosquitoes on potential host species and the hosts’ relative abundance.

From the originally retrieved 585 articles, 9 matched the inclusion criteria and were included in this review. All but one of the included studies were conducted in the Americas, six in the United States, and one each in Mexico and Colombia. The remaining study was conducted in Italy.

American Robin, Northern Cardinal, and House Finch were the most significantly preferred birds in the Americas, Common Blackbird in Italy.

**Conclusions/Significance:**

Although ornithophilic, all observed WNV-transmitting mosquitoes presented opportunistic feeding behaviour. All the observed species showed potential to act as bridges for zoonotic diseases, feeding also on humans. All the observed mosquitoes presented host selection patterns and did not feed on hosts as expected by chance alone.

The articles observe different species of mosquitoes in different environments. In addition, the way the relative host abundance was determined differed. Finally, this review is not systematic. Therefore, the translation of our results to different settings should be conducted cautiously.

## Author summary

West Nile virus (WNV) is a mosquito-borne virus that can cause a neuroinvasive and potentially deadly disease in vertebrates (including humans). The disease is transmitted from vectors (mostly *Culex* mosquitoes) during the blood meal. As no specific therapy or vaccination are available, the control of human cases centres on mosquitoes–humans contact prevention. For these reasons, deep knowledge of WNV transmission dynamics is considered paramount to focus preventive strategies. Nevertheless, WNV transmission dynamics are still unclear, especially regarding the role of potential hosts. Mosquitoes are intrinsically attracted from one host over another. However, in natural settings, absolute selection is often impossible. Therefore, in this review, we aimed at summarising knowledge on WNV-transmitting mosquitoes’ host selection in natural settings. We specifically considered original scientific publications calculating forage ratio for *Culex* mosquitoes. The forage ratio is the proportion of the relative abundance of blood meals taken from a potential host in trapped mosquitoes and that host’s relative abundance. Our results suggest that all observed *Culex* mosquitoes presented an opportunistic feeding behaviour and the potential to transmit zoonotic pathogens, since they all fed also on humans. Moreover, all the observed mosquitoes fed on hosts with a certain degree of selection, rather than what might be expected by host abundance alone.

## Introduction

West Nile virus (WNV) infections in humans are largely asymptomatic (ca. 80%), although 15% to 20% of infected humans develop the so-called West Nile fever, with unspecific symptoms such as headache, fever, and myalgia; and ca. 1% of infected humans develop the so-called West Nile neurological disease, with neurological conditions such as meningitis, encephalitis, and flaccid paralysis [1]. Among individuals who develop the neurological disease, mortality can be present, ranging from 4% to 14% [1]. Due to this high proportion of asymptomatic cases, low mortality rates, and initially limited geographic distribution to the Middle Eastern and Central African regions, WNV has been a largely neglected public health concern [1–5]. However, recent WNV outbreaks recorded since the mid- and late 1990s, together with the increasing number of confirmed human cases, have called for a different perspective on the pathogen [2,6–8]. Because of this rise in confirmed cases, WNV is now considered the most globally widespread arbovirus, and a major public health threat [3,9], with 52,532 confirmed human cases recorded in the United States (US) between 1999 and 2020 [10] and 2,663 cases in Europe between 2014 and 2019 [11–16].

Nevertheless, several aspects of the dynamics of the transmission of WNV are yet unclear, especially in terms of which vertebrate species are involved as hosts and to which extent [17]. WNV is maintained in nature in an enzootic cycle between mosquitoes (mainly from genus *Culex*) as vectors and certain birds as primary amplifying hosts [18,19]. Other vertebrates, especially mammals, can develop WNV infections. However, most of mammal hosts (e.g., most commonly humans and horses)—alongside several bird species—usually do not develop sufficient viremia to reinfect susceptible mosquitoes, thus presenting with low host competence for WNV infection (also known as dead-end hosts) [20,21].

Another key aspect, which influences the ability of vertebrate species as hosts, or their vertebrate host capacity, is the rate of contact with the vectors. The rate of contact is influenced both from feeding patterns of the mosquitoes, which are, in turn, based on specific characteristics, which the mosquitoes implement to find potential hosts, but also on the availability of the hosts [22–24]. More in detail, when defining feeding patterns of mosquitoes, two aspects are often considered: host preference and host selection [25,26]. The first is defined as the process of favouring and choosing a host among other equally available hosts, and it is based on intrinsic characteristics of the mosquitoes. The second is defined as the selection of hosts carried out from mosquitoes in nature, where not all hosts are equally available, and other factors such as climatic or environmental factors might play a role. This selection is still based on mosquitoes’ preferences but at the same time also on other ecological aspects (e.g., the availability of the hosts and the interactions with the mosquitoes) [25,26].

Whereas in order to prove host preference, experimental settings must be considered (e.g., baited traps) and equal access to different hosts must be provided, host selection has been tested in field conditions considering—among other strategies—the ratio between the blood meals and the availability of potential hosts, also called “forage ratio,” “feeding index,” “feeding preference,” “Index2,” or “selection index” [23]. In the case of studies on mosquito population, the forage ratio (often referred to as *w*_*i*_ or *P*_*i*_) for species *i* is defined as the ratio between the proportion of blood meals originated on the specie *i* and the relative abundance of the specie *i* in the environment.

This index is then interpreted as follows: a value equal to 1 shows no selection but rather the feeding pattern that could be expected if feeding was a function of chance alone. Conversely, a value greater than 1 can be interpreted as feeding on the species *i* more than what might be expected by chance alone (preference), whereas a value lower than 1 as the opposite (avoidance) [22,26].

The knowledge of the host selection patterns of the different mosquito species in a given geographical area can help—on one side—to identify which hosts are more important in the transmission of the virus, while on the other side, it can help to evaluate the potential of the specific mosquito species to act as a bridge between different vertebrate species—in this case, also humans—allowing the transmission of zoonotic and pandemic pathogens [27–29]. In fact, as the virus is transmitted with the blood meal, feeding or not on a specific host will determine the risk of this host to develop the disease. For this reason, understanding which mosquito species feed on humans, and to which extent, would allow to better evaluate the risk of transmission of a certain disease to humans, in specific geographical areas and different climatic conditions. Similarly, identifying host species with major amplification role in the transmission of the disease might allow for more focused control strategies [24,30].

We considered these aspects to be relevant as, to date, no vaccination or specific therapy is available for human WNV infections. Hence, preventive strategies are the only tool at disposal to control the incidence of the disease in humans. In addition, considering the role that climatic factors exercise on the mosquitos’ abundance and vector ability, prevention strategies are crucial also in addressing the future risk of transmission in geographical areas, which are affected from changes in climatic conditions. Therefore, in order to focus these preventive strategies, gaining more understanding of the specific aspects of the WNV transmission dynamics should be considered of paramount importance, especially in relation to which vectors and hosts are involved and to which extent [23,24,31,32].

In this article, we aimed to explore the patterns of host selection of WNV-transmitting *Culex* mosquitoes. We summarized the scientific evidence of preference or avoidance of avian and mammalian hosts by *Culex* mosquitoes, in the different geographical settings. The results of this review suggest that WNV-transmitting *Culex* mosquitoes, even when largely ornithophilic, present with opportunistic feeding behaviour, feeding both on avian and mammals host (including humans). The presence of blood meals taken on human suggests that all observed mosquitoes have the potential of transmitting zoonotic pathogens to humans. In addition, all the observed mosquitoes showed a certain degree of selection of the potential hosts, not feeding as it would have been expected from the relative abundance of potential hosts alone.

## Methods

### Search strategy

In July 2022, we searched the PubMed online database for original studies with the following search strategy:

((blood[Title/Abstract]) OR (host*[Title/Abstract]) OR (selection[Title/Abstract]) OR (preference*[Title/Abstract]) OR (feed*[Title/Abstract])) AND ((Culex[MeSH Terms]) OR (Culex[Title/Abstract])) AND ((West Nile virus[MeSH Terms]) OR (West Nile [Title/Abstract]) OR (WNV[Title/Abstract])).

The resulting articles were exported for further screening to EndNote X9 (Clarivate Analytic, Philadelphia, PA, USA).

### Inclusion/exclusion criteria

We screened the articles first on a title-and-abstract level and after on a full-text level. In order to correctly represent host selection, the screening was conducted based on the following inclusion criteria:

- Original field studies and
- Studies estimating forage ratio

Based on these criteria, we excluded non-original studies (e.g., reviews, comments, and letters to the editors) and non-field studies (e.g., models, laboratory-based studies). In addition, we excluded studies, which did not consider birds (due to their major role in WNV transmission), aimed to estimate mosquitoes’ host preferences (e.g., using selected species), considering only large groups of possible hosts (e.g., “avian,” “human,” or “mammals”), as well as a single host species versus all the other pooled ones. Finally, we excluded studies that aimed to explore the presence of WNV-specific antibodies in potential host species.

Furthermore, in order to obtain comparable results, studies that reported out-of-date techniques for blood meal analysis (e.g., precipitin test) were excluded.

No limitations in terms of year of publication or language of the article were considered.

### Data extraction and presentation

Considering the explorative nature of this study as well as the intrinsic level of difference among studies on host selection and forage ratio, no systematic approach to the review was conducted. One author (NR) screened the articles retrieved by the search and extracted the ones to include. The following information on the included studies were extracted: first author and year of publication, country, urban/rural settings, time frame, number of study sites, strategy to collect mosquitoes, mosquitoes species observed, number of collected mosquitoes, success of blood meals identification, identified host species (inclusive humans), and strategy to evaluate host abundance.

The relative proportion of blood meals taken on the 10 most common host species in each of the observed mosquito species in the included articles was reported. When the proportion of blood meals taken was reported only separately for different host subgroups (e.g., “avian” and “non-avian”), we calculated and reported the proportion over all observed hosts. In addition, the estimated forage ratios (which we reported as *w*_*i*_ or *P*_*i*_, based on the original definition in each article) were reported alongside the specific standard error (SE), confidence interval (CI), or *p*-value (*p*) calculated in each paper, for statistical significance of the estimate. To enhance readability, forage ratios were presented stratified for taxonomic order of the observed hosts. The order Passeriformes was presented independently from the others for two main reasons: first, the absolute abundance of bird species within the order and, second, the specific impact in the transmission of WNV. In fact, birds from the order Passeriformes are often considered to be particularly involved in WNV enzootic cycle, both for their availability as hosts and for their assumed level of WNV viremia, and, therefore, host competence [20].

## Results

### Included articles

The research retrieved overall 585 records, while title and abstract screening returned 92 articles. After full-text screening, 9 articles matched our inclusion criteria and were considered for this review [22,33–40]. One article was retrieved, which calculated forage ratio for a single potential host species (American Robin) together with the pooled forage ratio for all other species [41]. For this reason, the article was not included in the main review.

All but one of the nine selected studies were conducted in the Americas. Six were conducted in the US (two in the urban area of Chicago, IL; one in the urban area of Las Vegas, NV; one in East Baton Rouge Parish, LA; one in the rural area of Davis, CA; and one in Maryland and Washington, DC) [22,33,36,37,39,40]. The other two were conducted in Colombia and Mexico [34,38]. The remaining study was conducted in the Northern Italian region Veneto [35].

Three studies considered multiple mosquito species (Kothera and colleagues [37]: *Cx*. *pipiens* complex and *Cx*. *restuans*; and both Hamer and colleagues [22] and Kilpatrick and colleagues [36]: *Cx*. *pipiens* and *Cx*. *restuans*, althought the latter study calculated a pooled forage ratio). Three studies considered a single species of mosquitoes (Hannon and colleagues [33]: *Cx*. *quinquefasciatus*; Thiemann and colleagues [39]: *Cx*. *tarsalis*; Mendenhall and colleagues [34]: *Cx*. *erraticus*). The study by Mackay and colleagues [40] collected data on three *Culex* species (*Cx*. *quinquefasciatus*, *Cx*. *Nigripalus*, and *Cx*. *salinaris*) but calculated the forage ratio only for *Cx*. *quinquefasciatus*. Similarly, the study by Rizzoli and colleagues [35] collected data on different species of mosquitoes but calculated the feeding index only for *Cx*. *pipiens*. The study by Estrada-Franco and colleagues [38] calculated the forage ratio for *Ae*. *aegypti* and *Cx*. *quinquefasciatus*. However, as our search focussed specifically on *Culex* mosquitoes, we reported only the results for the latter species. Conversely, the study by Thiemann and colleagues [39] calculated forage ratios only for *Cx*. *tarsalis*, but in two different seasons (late summer and winter) (Table 1).

**Table 1:** Characteristics of the included studies.

First author, year of publication	Place and country	Urban or rural setting	Data collection time frame	Collection sites	Evaluation of potential host abundance	Mosquitoes species observed*	Mosquitoes collection strategies	Mosquitoes collected*	Blood meals identified (tested)*	Host species identified
Kothera, L., 2020 [37]	Chicago, IL (USA)	Urban (Residential)	July–August, 2012	8 residential, 1 public park	Point count survey	*Cx*. *pipiens* complex	Backpack aspirators	887	840 (1,023)	15 avian, 3 mammalian (incl. humans)
						*Cx*. *restuans*		72		
Hannon, E.R., 2019 [33]	Las Vegas, NV (USA)	Urban	August–September, 2016	76	Citizen science data (eBirds.org)	*Cx*. *quinquefasciatus*	Gravid trap	2,462	164 (195)	19 avian, 3 mammalian (incl. humans)
Thiemann, T.C., 2011 [39]	Davis, CA (USA)	Rural (Farmstead)	May–October, 2008 (late summer) and January–June 2009 (winter)	3 (+2 control)	Point count survey	*Cx*. *tarsalis*	Dry-ice baited CDC-style traps and aspirator	NA	851 (925)	29 avian, 7 mammalian (incl. humans), and 1 reptile
Hamer, G.L., 2009 [22]	Chicago, IL (USA)	Suburban	May—October, 2005–07	11 residential, 4 semi-natural (2005); 21 residential, 5 semi-natural (2006); 15 residential, 4 natural (2007)	Point count survey and captures	*Cx*. *pipiens*	CDC light traps, gravid traps, and backpack aspirators	1,483	1,043 (1,483)	25 avian, NA mammalian
						*Cx*. *restuans*				18 avian, NA mammalian
Mendenhall, I.H., 2012 [34]	Sonso Lake, Cauca Valley, (CO)	Rural	July—August, 2008	Each avian census point (16) and additional 5	Point count survey	*Cx*. *erraticus*	Backpack aspirators, resting boxes	1,515	NA	9 avian, 5 mammalian (incl. humans), and 1 reptilian
Estrada-Franco, J.G., 2020 [38]	Reynosa, Tamaulipas (MX)	Semi-urban	May–July, 2018 and September–November, 2018	16 households	Point count survey and questionnaire census	*Cx*. *quinquefasciatus*	Resting boxes, CDC backpack aspirators, BG sentinel traps and AGO traps	10,711	388 (475)	11 avian, 9 mammalian (incl. humans), and 1 reptilian
Mackay, A.J., 2010 [40]	East Baton Rouge Parish, LA (USA)	Urban, sub-urban and rural	November 2002-October 2004	18	North American Breeding Bird Survey (NABBS) 2003–2004	*Cx*. *quinquefasciatus*	CDC light traps, and CDC gravid traps	NA	NA (1,097)	47 avian and 16 mammalian
Kilpatrick, A.M., 2006 [36]	Maryland and Washington, DC (USA)	Urban and residential	May–September, 2004	3 urban, 2 residential	Point count survey	*Cx*. *pipiens* *Cx*. *restuans*	CDC light traps, CDC gravid traps, and backpack aspirators	Approx. 23,000	163 (181)	NA
Rizzoli, A., 2015 [35]	Veneto Region, Italy	Rural and peridomestic	May–October, 2012	10 rural, 1 peridomestic	Point count survey	*Cx*. *pipiens*	BG sentinel traps	206	188 (206)	31 wild avian species

*Reported are only the absolute numbers of mosquitoes considered for this review. Absolute numbers of mosquitoes from other species collected or tested during the study were not reported.

### Blood meal analysis

American Robin (*Turdus migratorius*) was the most common blood meal host in the studies by Kothera and colleagues [37] (41.5% of blood meals taken by *Cx*. *pipiens* complex mosquitoes) and by Hamer and colleagues [22] (39.2% and 37.6% of blood meals taken by *Cx*. *pipiens* and *Cx*. *restuans*, respectively). In addition, it was the second most common blood meal host in the study by Thiemann and colleagues [39] (16% of blood meals taken by *Cx*. *tarsalis* in winter) and the third in the study by Hannon and colleagues [33] (7.9% of blood meals taken by *Cx*. *quinquefasciatus*). Although the article did not present a list of the most common blood meal hosts, American Robin accounted for the 43% ± 9% of blood meals taken by *Cx. pipiens* and *Cx*. *restuans* in the work by Kilpatrick and colleagues [36].

House Sparrow (*Passer domesticus*) was the most common blood meal host in the study by Kothera and colleagues [37] (38.9% of blood meals taken by *Cx*. *restuans*). In addition, it was the second most common in the studies by Kothera and colleagues [37] (26.3% of all blood meals taken by *Cx*. *pipiens* complex) and Thiemann and colleagues [39] (16% of all blood meals taken by *Cx*. *tarsalis* in winter). Blood meals from House Sparrows were among the 10 most common blood meals in the studies by Hamer and colleagues [22] (11.9% and 14.0% of blood meals taken by *Cx*. *pipiens* and *Cx*. *restuans*, respectively), Hannon and colleagues [33] (6.7% of blood meals taken by *Cx*. *quinquefasciatus*), and Thiemann and colleagues [39] (1% of blood meals taken by *Cx*. *tarsalis* in late summer). In the paper by Kilpatrick and colleagues [36], House Sparrow accounted for 11% ± 4% of the mosquito feedings.

House Finch (*Haemorhous mexicanus*) was the most common blood meal host in the study by Hannon and colleagues [33] (38.4% of blood meals taken by *Cx*. *quinquefasciatus*). In addition, it was among the 10 most common blood meal host in the studies by Kothera and colleagues [37] (7.4% and 2.8% of blood meals taken by *Cx*. *pipiens* complex and *Cx*. *restuans*, respectively), Thiemann and colleagues [39] (7% of blood meals taken by *Cx*. *tarsalis* in winter), and Hamer and colleagues [22] (5.3% and 3.6% of blood meals taken by *Cx*. *pipiens* and *Cx*. *restuans*, respectively).

Other commonly reported blood meal hosts were Cedar Waxwing (*Bombycilla cedrorum*) [37], Mourning Dove (*Zenaida macroura*) [22,33,38–40], and Northern Cardinal (*Cardinalis cardinalis*) [22,40].

Although the majority of blood meals reported were taken on birds, opportunistic feeding behaviour from the mosquitoes—in the form of blood meals taken also on mammals—was reported in all the included studies [22,33–40]. Furthermore, the potential of acting as bridge for zoonotic diseases—in the form of blood meals taken on humans—was reported in all the included studies. Blood meals taken on humans were especially common in the studies by Hamer and colleagues [22], Mendenhall and colleagues [34], and Estrada-Franco and colleagues [38] (15.7% and 14.0% of blood meals taken by *Cx*. *pipiens* and *Cx*. *restuans*, respectively; and 17.3% of blood meals taken by *Cx*. *erraticus*; 3.5% of blood meals taken by *Cx*. *quinquefasciatus*) (Table 2).

**Table 2:** Relative frequency of the 10 most common blood meals for each observed mosquito species in the included studies.

Rank	Kothera, 2020 [37]				Hannon, 2019 [33]		Thiemann, 2011 [39]				Hamer, 2009* [22]				Mendenhall, 2012 [34]		Estrada-Franco, 2020 [38]		Mackay, 2010 [40]	
	*Cx*. *pipiens* complex		*Cx*. *restuans*		*Cx*. *quinquefasciatus*		*Cx*. *tarsalis—Late summer*		*Cx*. *tarsalis—winter*		*Cx*. *pipiens*		*Cx*. *restuans*		*Cx*. *erraticus*		*Cx*. *Quinquefasciatus*		*Cx*. *quinquefasciatus*	
	Species	%	Species	%	Species	%	Species	%	Species	%	Species	%	Species	%	Species	%	Species	%	Species	%
1	American Robin	41.5	House Sparrow	38.9	House Finch	38.4	Black-crowned Night-Heron	69	Yellow-billed Magpie	43	American Robin	39.2	American Robin	37.6	Limpkin	20.6	Domestic Dog	66.8	Northern Cardinal	14.9
2	House Sparrow	26.3	American Robin	33.3	Domestic Chicken	16.5	Domestic Cow	10	American Robin	16	Human	15.7	Human	14.0	Human	17.3	Domestic Chicken	6.8	Northern Raccoon	11.5
3	House Finch	7.4	Northern Cardinal	6.9	American Robin	7.9	Snowy Egret	7	House Sparrow	16	House Sparrow	11.9	House Sparrow	14.0	Black-crowned Night-Heron	14.8	House Sparrow	4.0	Northern Mockingbird	9.1
4	Cedar Waxwing	4.6	Cedar Waxwing	4.2	House Sparrow	6.7	Mourning Dove	4	House Finch	7	Mourning Dove	8.6	Northern Cardinal	8.6	Striated Heron	10.3	Human	3.5	Domestic Dog	8.3
5	Mourning Dove	3.6	Cooper’s Hawk	4.2	Great-tailed Grackle	6.1	Great Egret	4	Red-tailed Hawk	7	Northern Cardinal	6.8	European Starling	5.0	Capybara	9.1	Mourning Dove	2.9	Human	7.0
6	Northern Cardinal	2.4	House Finch	2.8	Northern Mockingbird	5.5	House Sparrow	1	Mourning Dove	6	House Finch	5.3	Mourning Dove	4.5	Cocoi Heron	6.2	Virginia Opossum	2.6	Common Grackle	6.3
7	Cooper’s Hawk	1.0	Mourning Dove	2.8	Mourning Dove	4.9	Domestic Goat	1	Several species	1	Blue Jay	2.2	House Finch	3.6	Iguana	4.9	Eurasian Collared-Dove	2.2	Virginia Opossum	6.0
8	Domestic Chicken	0.6	Several species	1.4	Rock Pigeon	3.0	Domestic Dog	1			European Starling	1.9	Red-winged Blackbird	1.8	Domestic Cow	2.9	Domestic Cat	1.8	Mourning Dove	4.8
9	European Starling	0.4			Brewer’s Sparrow	1.8	Several species	<1			Northern Raccoon	1.6	Northern Raccoon	1.4	Bare-faced Ibis	2.5	Rock Dove	1.8	Domestic Chicken	4.1
10	Baltimore Oriole and American Kestrel	0.2			Lazuli Bunting and Turkey Vulture	1.2					Several species	0.5	Common Grackle and Scarlet Tanager	1.4	Least Bittern	2.1	Wild Turkey	1.8	House Sparrow	3.2

*The proportion of the blood meals was reported only divided by host class (e.g., “avian” and “non-avian”) in the study. For comparability with other studies, here were reported as proportion of all hosts.

** Studies by Kilpatrick and colleagues [36] and Rizzoli and colleagues [35] did not provide with detailed information on the proportion of blood meals from the single hosts species. For this reason, they were not included in this table

### Preference and avoidance of hosts

Among Passeriformes, American Robin (*Turdus migratorius*) was significantly preferred by *Cx*. *pipiens* complex (*w*_*i*_ = 3.40, SE = 0.43; [37]), *Cx*. *pipiens* (*w*_*i*_ = 2.26, SE = 0.39; [22]), and *Cx*. *restuans* in Chicago, IL (USA) (*w*_*i*_ = 1.80, SE = 0.39 according to Kothera and colleagues [37]; and *w*_*i*_ = 1.92, SE = 0.36 according to Hamer and colleagues [22]); by *Cx*. *pipiens* and *Cx*. *restuans* in Maryland and Washington, DC (USA) (*w*_*i*_ = 16.7 ± 4.4; [36]); by *Cx*. *quinquefasciatus* in Las Vegas, NV (USA) (forage ratio = 38.42, 95% CI 16.90, 82.09); and by *Cx*. *tarsalis* in Davis, CA (USA) (*w*_*i*_ = 27.71, SE = 13.66 in winter; [39]). In the only non-American article, the Common Blackbird (*Turdus merula*) was preferred by *Cx*. *pipiens* in North-Eastern Italian region Veneto (*P*_i_ = 8.25, *p* < .001) [35].

In addition, Northern Cardinal (*Cardinalis cardinalis*) was also significantly preferred by *Cx*. *pipiens* complex (*w*_*i*_ = 3.85, SE = 2.37; [37]), *Cx*. *pipiens* (*w*_*i*_ = 5.5, SE = 3.87; [22]), and *Cx*. *restuans* in Chicago, IL (USA) (*w*_*i*_ = 7.38, SE = 5.33 according to Kothera and colleagues [37]; and *w*_*i*_ = 6.20, SE = 4.48 according to Hamer and colleagues [22]); and by *Cx*. *quinquefasciatus* in Reynosa, Tamaulipas (Mexico) (*w*_i_ = 2.90, 95% CI 2.50, 3.30; [38]).

House Finch (*Haemorhous mexicanus*) in Chicago, IL, and Las Vegas, NV (USA) [22,33], Northern Mockingbird (*Mimus polyglottos*) in Las Vegas, NV (USA) and in Reynosa, Tamaulipas (Mexico) [33,38], Brown Trasher (*Toxostoma rufum*) in Reynosa, Tamaulipas (Mexico) [38], and Yellow-billed Magpie (*Pica nuttalli*) in Davis, CA (USA) [39] were all significantly preferred by at least one species of mosquitoes. Similarly, Magpies (*Pica pica*) were preferred by *Cx*. *pipiens* in Veneto (Italy) [35].

Conversely, American Crow (*Corvus brachyrhynchos*) was avoided by *Cx*. *pipiens* complex (*w*_*i*_ = 0.05, SE = 0.05; [37]) and *Cx*. *restuans* in Chicago, IL (USA) (*w*_*i*_ = 0.37, SE = 0.38; [37]). American Goldfinch (*Spinus tristis*) was avoided by *Cx*. *pipiens* complex (*w*_*i*_ = 0.02, SE = 0.02; [37]), *Cx*. *pipiens* (*w*_*i*_ = 0.09, SE = 0.01; [22]), and *Cx*. *restuans* in Chicago, IL (USA) (*w*_*i*_ = 0.16, SE = 0.16 according to Kothera and colleagues [37]; and *w*_*i*_ = 0.22, SE = 0.25 according to Hamer and colleagues [22]). Similarly, European Starling (*Sturnus vulgaris*) was avoided by *Cx*. *pipiens* complex (*w*_*i*_ = 0.04, SE = 0.02; [37]) and *Cx*. *restuans* in Chicago, IL (USA) (*w*_*i*_ = 0.09, SE = 0.09; [37]); by *Cx*. *pipiens* (*w*_*i*_ = 0.39, SE = 0.17 in Chicago, IL (USA), according to Hamer and colleagues [22]; and *P*_*i*_ = 0.09, *p* < .001 in Veneto (Italy), according to Rizzoli and colleagues [35]), and by *Cx*. *tarsalis* in winter in Davis, CA (USA) (*w*_*i*_ = 0.05, SE = 0.05; [39]).

House Sparrow (*Passer domesticus*) was significantly avoided by *Cx*. *pipiens* complex (*w*_*i*_ = 0.90, SE = 0.08; [37]), *Cx*. *pipiens* (*w*_*i*_ = 0.32, SE = 0.05; [22]), and *Cx*. *restuans* in Chicago, IL (USA) (*w*_*i*_ = 0.33, SE = 0.06; [22]); by *Cx*. *pipiens* and *Cx*. *restuans* in Maryland and Washington, DC (USA) (*w*_*i*_ = -7.9 ± 2.5; [36]); by *Cx*. *tarsalis* in Davis, CA (USA) (*w*_*i*_ = 0.13, SE = 0.09 and *w*_*i*_ = 0.44, SE = 0.10 in late summer and winter, respectively; [39]), and *Cx*. *quinquefasciatus* in Reynosa, Tamaulipas (Mexico) (*w*_*i*_ = 0.50, 95% CI 0.30, 0.60; [38]). However, according to Hannon and colleagues [33], House Sparrow was significantly preferred by *Cx*. *quinquefasciatus* in Las Vegas, NV (USA) (*w*_*i*_ = 4.74, 95% CI 2.37, 9.19), while according to Rizzoli and colleagues [35], it was utilized in the same proportion as if feeding was based on chance alone in Veneto (Italy) (*P*_*i*_ = 1.01, *p* < .05). Similarly, Common Grackle (*Quiscula quiscula*) was significantly avoided by *Cx*. *pipiens* complex (*w*_*i*_ = 0.29, SE = 0.35; [37]), *Cx*. *pipiens* (*w*_*i*_ = 0.06, SE = 0.05; [22]), and *Cx*. *restuans* in Chicago, IL (USA) (*w*_*i*_ = 0.24, SE = 0.16; [22]), but significantly preferred by *Cx*. *quinquefasciatus* in Reynosa, Tamaulipas (Mexico) (*w*_*i*_ = 1.60, 95% CI 1.20, 2.00; [38]) (Table 3).

**Table 3:** Forage ratio for birds of the order Passeriformes in the selected studies. A result equal to 1 represents opportunistic feeding behaviour, while one greater than 1 or lower than1 represents preference and avoidance, respectively. Bird species are reported in alphabetic order. Species that are significantly preferred and/or avoided are reported in bold.

Bird species	Kothera, 2020 [37]		Hannon, 2019 [33]	Thiemann, 2011 [39]		Hamer, 2009 [22]		Mendenhall, 2012 [34]	Estrada-Franco, 2020 [38]		Kilpatrick, 2006 [36]	Rizzoli, 2015 [35]
	*Cx*. *pipiens* complex	*Cx*. *restuans*	*Cx*. *quinquefasciatus*	*Cx*. *tarsalis—Late summer*	*Cx*. *tarsalis—Winter*	*Cx*. *pipiens*	*Cx*. *restuans*	*Cx*. *erraticus*	*Cx*. *quinquefasciatus*	*Cx*. *quinquefasciatus*	*Cx*. *pipiens and Cx*. *restuans*	*Cx*. *pipiens*
*American Crow*	**0.05**	**0.37**				0.54	1.37					
*American Goldfinch*	**0.02**	**0.16**				**0.09**	**0.22**					
*American Redstart*	**0.08**	0.63										
*American Robin*	**3.40**	**1.80**	**38.42**		**27.71**	**2.26**	**1.92**				**16.70**	
*Acadian Flycatcher*	0.58	4.43										
*Baltimore Oriole*	1.15	4.43				1.12	2.85					
*Barn Swallow*						1.94	4.96					**0.02**
*Black-and-White Warbler*	0.58	4.43										
*Black-capped Chickadee*	**0.05**	**0.40**				1.86	2.37					
*Blue Jay*	0.58	4.43				8.44	3.08			0.90		
*Blue-gray Gnatcatcher*						1.81	4.62					
*Brewer’s Sparrow*			4.83									
*Brown Trasher*						24.17				**2.50**		
*Brown-headed Cowbird*			2.00			0.65	3.34					
*Canada Warbler*	0.58	4.43										
*Cedar Waxwing*	22.50	13.29				0.59	0.75					
*Chipping Sparrow*						0.46	0.59					
*Cliff Swallow*	0.58	4.43										
*Common Blackbird*												**8.25**
*Common Canary*						25.17						
*Common Grackle*	**0.29**	2.22				**0.06**	**0.24**			**1.60**		
*Common Raven*			5.24									
*Common Redpoll*	0.58	4.43										
*Common Yellowthroat*						6.95	17.73					
*Eastern Bluebird*						10.75	27.43					
*Eastern Kingbird*						4.04	10.32					
*Eastern Towhee*						11.85	30.24					
*Eastern Wood-pewee*	0.58	4.43				2.67	6.8					
*European Starling*	**0.04**	**0.09**			**0.05**	**0.39**	0.91					**0.89**
*Field Sparrow*							64.25					
*Fish Crow*										0.70	24.60 and 10.40*	
*Gray Catbird*	0.58	4.43				0.78	1.99					
*Great Crested Flycatcher*	0.58	4.43				3.64	9.28					
*Great-tailed Grackle*			0.89									
*House Finch*	35.77	8.86	**7.65**	0.99	1.17	**5.69**	3.42					
*House Martin*												0.14
*House Sparrow*	**0.90**	0.87	**4.74**	**0.13**	**0.44**	**0.32**	**0.33**		0.22	**0.50**	**−7.90**	1.01
*House Wren*						1.82	1.55					
*Indigo Bunting*						3.09	7.89					
*Lazuli Bunting*			2.35									
*Lesser Goldfinch*			1.09									
*Lincoln’s Sparrow*					7.29							
*Magpie*												**3.54**
*Northern Cardinal*	**3.85**	**7.38**				**5.50**	**6.20**			**2.90**		
*Northern Mockingbird*			**3.34**							**1.80**		
*Prothonotary Warbler*	0.58	4.43										
*Red-eyed Vireo*	**0.29**	2.22				2.49	6.35					
*Red-winged Blackbird*						**0.08**	**0.41**					
*Scarlet Tanager*						34.09	87.00					
*Song Sparrow*						2.11	2.69					
*Swainson’s Thrush*	0.19	1.48				50.34						
*Swamp Sparrow*							64.25					
*Tufted Titmouse*										2.00		
*Veery*						3.12	7.97					
*Warbling Vireo*	0.58	4.43				3.54	9.02					
*Western Scrub-Jay*					7.29							
*Western Tanager*			2.33									
*White-breasted Nuthatch*	0.58	4.43				3.29	8.39					
*Willow Flycatcher*						7.17	18.30					
*Yellow Warbler*	0.58	4.43				2.27	5.80					
*Yellow-billed Magpie*					**22.79**							
*Yellow-rumped Warbler*	0.58	4.43			**0.05**							

*Results from 2 different sites.

Among non-Passeriformes, Turkey Vulture (*Cathartes aura*) and Limpkin (*Aramus guarana*) were significantly preferred (*w*_*i*_ = 4.01, 95%CI 1.06, 15.17 and *w*_*i*_ = 13.31, SE = 1.64 for *Cx*. *quinquefasciatus* in Las Vegas, NV [USA] and *Cx*. *erraticus* in Sonso Lake, Cauca Valley [Colombia], respectively; [33,34]). Black-crowned Night-Heron (*Nycticorax nycticorax*) was preferred by *Cx*. *tarsalis* in Las Vegas, NV (USA) and *Cx*. *erraticus* in Sonso Lake, Cauca Valley (Colombia) (*w*_*i*_ = 1.32, SE = 0.07, and *w*_*i*_ = 21.88, SE = 3.21, respectively; [34,39]). Conversely, Canada Goose (*Branta canadensis*; *w*_*i*_ = 0.03, SE = 0.04 and *w*_*i*_ = 0.26, SE = 0.27 for *Cx*. *pipiens* complex and *Cx*. *restuans*, respectively, in Chicago, IL [USA]; [22,37]) and Chimney Swift (*Chaetura pelagica*; *w*_*i*_ = 0.04, SE = 0.04 and *w*_*i*_ = 0.32, SE = 0.33 for *Cx*. *pipiens* complex and *Cx*. *restuans*, respectively, in Chicago, IL [USA]; [37]) were both avoided. In addition, Ring-billed Gull (*Larus delawarensis*; *w*_*i*_ = 0.10, SE = 0.10 for *Cx*. *pipiens* complex in Chicago, IL [USA]; [37]) and Rock Pigeon (*Columba livia*; *w*_*i*_ = 0.01, SE = 0.01 and *w*_*i*_ = 0.11, SE = 0.11 for *Cx*. *pipiens* complex and *Cx*. *restuans* in Chicago, IL [USA]; [37]; and *w*_*i*_ = 0.20, 95% CI 0.08, 0.46 for *Cx*. *quinquefasciatus* in Las Vegas, NV [USA] [33]; *w*_*i*_ = 0.19, SE = 0.25 and *P*_*i*_ = 0.34, *p* < .001 for *Cx*. *pipiens* according to Hamer and colleagues [22] in Chicago, IL [USA] and Rizzoli and colleagues [35] in Veneto [Italy], respectively) were also avoided. Similarly, Monk Parakeet (*Myopsitta monachus*; *w*_*i*_ = 0.29, SE = 0.35 for *Cx*. *pipiens* complex in Chicago, IL [USA]; [37]), Red-bellied Woodpecker (*Melanerpes carolinus*; *w*_*i*_ = 0.29, SE = 0.35 for *Cx*. *pipiens* complex in Chicago, IL [USA]; [37]), and Downy Woodpecker (*Picoides pubescens*; *w*_*i*_ = 0.29, SE = 0.35 for *Cx*. *pipiens* complex in Chicago, IL [USA]; [37]) were all avoided. Snowy Egret (*Egretta thula*) was avoided by *Cx*. *tarsalis* in Sonso Lake, Cauca Valley (Colombia) and *Cx*. *erraticus* in Davis, CA (USA) (*w*_*i*_ = 0.32, SE = 0.10 and *w*_*i*_ < 0.08, SE = 0.08, respectively; [34,39]) (Table 4).

**Table 4:** Forage ratio for birds other than Passeriformes in the selected studies. A result equal to 1 represents opportunistic feeding behaviour, while one greater than 1 or lower than 1 represents preference and avoidance, respectively. Bird species are reported in alphabetic order. Species that are significantly preferred and/or avoided are reported in bold. Species are reported divided by order.

Bird species	Kothera, 2020 [37]		Hannon, 2019 [33]	Thiemann, 2011 [39]		Hamer, 2009 [22]		Mendenhall, 2012 [34]	Estrada-Franco, 2020 [38]	Mackay, 2010 [40]	Kilpatrick, 2006 [36]	Rizzoli, 2015 [35]
	*Cx*. *pipiens* complex	*Cx*. *restuans*	*Cx*. *quinquefasciatus*	*Cx*. *tarsalis*—Late summer	*Cx*. *tarsalis*–Winter	*Cx*. *pipiens*	*Cx*. *restuans*	*Cx*. *erraticus*	*Cx*. *quinquefasciatus*	*Cx*. *quinquefasciatus*	*Cx*. *pipiens and Cx*. *restuans*	*Cx*. *pipiens*
Order: Accipitriformes												
*American Kestrel*	1.15	4.43	7.67			75.50						
*Cooper’s Hawk*	4.62	13.29				25.17						
*Red-tailed Hawk*					58.34							
*Snail Kite*								**<0.12**				
*Turkey Vulture*			**4.01**									
Order: Anseriformes												
*Canada Goose*	**0.03**	**0.26**										
*Mallard*						**0.20**	0.51		2.47			
*Muscovy Duck*												
Order: Apodiformes												
*Chimney Swift*	**0.04**	**0.32**										
Order: Charadriiformes												
*Killdeer*						4.68	11.94					
*Ring-billed Gull*	**0.10**	0.74										
Order: Columbiformes												
*Eurasian Collared-Dove*						4.65	11.86		0.50			1.36
*Inca Dove*									0.09			
*Mourning Dove*	0.91	**0.47**	0.81	**69.00**	**4.25**	**1.55**	0.80		3.92	1.40		
*Rock Pigeon*	**0.01**	**0.11**	**0.20**			**0.19**	0.49		1.61			**0.34**
Order: Cuculiformes												
*Greater Roadrunner*			8.41									
*Smooth-billed Ani*								**<0.13**				
Order: Galliformes												
*Domestic Chicken*	2.89	4.43							1.01			
*Gambel’s Quail*			0.27									
*Ring-necked Pheasant*						25.17	64.25					
*Wild Turkey*									4.83			
Order: Gruiformes												
*Common Moorhen*								**0.09**				
*Limpkin*								**13.31**				
Order: Pelecaniformes												
*Bare-faced Ibis*								**0.35**				
*Black-crowned Night-Heron*				**1.32**				**21.88**				
*Cattle Egret*				**0.15**				**<0.10**				
*Cocoi Heron*								**7.62**				
*Great Egret*				0.85				**<0.07**				
*Pinnated Bittern*								8.95				
*Snowy Egret*				**0.32**				**<0.08**				
*Striated Heron*								**7.71**				
Order: Piciformes												
*Downy Woodpecker*	**0.29**	2.22				0.53	1.35					
*Hairy Woodpecker*						12.96	33.08					
*Northern Flicker*	0.58	4.43				5.40	13.78					
*Red-bellied Woodpecker*	**0.29**	2.22										
Order: Psittaciformes												
*Blue-headed Parrot*								**<0.13**				
*Monk Parakeet*	**0.29**	2.22				**0.11**	**0.28**					
Order: Strigiformes												
*Barn Owl*				19.71	7.29							
*Great Horned Owl*				19.71	7.29							
Order: Suliformes												
*Anhinga*								4.97				
*Neotropic Cormorant*												

Among non-avian species, Domestic Cows (*Bos taurus*) and Domestic Dogs (*Canis lupus familiaris*) were significantly preferred by *Cx*. *tarsalis* in late summer in Davis, CA (USA) (*w*_*i*_ = 52.57, SE = 24.82; and *w*_*i*_ = 9.86, SE = 8.50, respectively; [39]). Domestic Dogs and Virginia Opossum (*Didelphis virginiana*) were preferred by *Cx*. *quinquefasciatus* in Reynosa, Tamaulipas (Mexico), but no level of significance was estimated in the study [38]. Domestic Cows were avoided by *Cx*. *erraticus* in Sonso Lake, Cauca Valley (Colombia) (*w*_*i*_ = 0.45, SE = 0.16) [34].

Humans were preferred by *Cx*. *erraticus* in Sonso Lake, Cauca Valley (Colombia) (*w*_*i*_ = 5.08, SE = 0.70) [34] and avoided by *Cx*. *quinquefasciatus* in Reynosa, Tamaulipas (Mexico), again with no level of significance estimated [38] (Table 5).

**Table 5:** Forage ratio for non-avian species in the selected studies. A result equal 1 represents opportunistic feeding utilization as for chance, while one greater than 1 or lower than 1 represents preference and avoidance, respectively. Host species are reported in alphabetic order. Species that are significantly preferred and/or avoided are reported in bold.

*Non-bird species*	Kothera, 2020 [37]		Hannon, 2019 [33]	Thiemann, 2011 [39]		Hamer, 2009 [22]		Mendenhall, 2012 [34]	Estrada-Franco, 2020 [38]	Mackay, 2010 [40]	Kilpatrick, 2006 [36]	Rizzoli, 2015 [35]
	*Cx*. *pipiens* complex	*Cx*. *restuans*	*Cx*.	*Cx*. *tarsalis*—Late summer	*Cx*. *tarsalis*—Winter	*Cx*. *pipiens*	*Cx*. *restuans*	*Cx*. *erraticus*	*Cx*. *quinquefasciatus*	*Cx*. *quinquefasciatus*	*Cx*. *pipiens and Cx*. *restuans*	*Cx*. *pipiens*
			*quinquefasciatus*									
*Capybara*								**12.30**				
*Domestic Cat*									0.20			
*Domestic Cow*				**52.57**				**0.45**				
*Domestic Dog*				**9.86**					5.64			
*Domestic Goat*				0.74								
*Domestic Horse*				6.57				0.99	0.52			
*Domestic Pig*									0.80			
*Texas Tortoise*									0.40			
*Virginia Opossum*									4.83			
*Human*								**5.08**	0.24			

## Discussion

A thorough understanding of virus transmission dynamics is paramount to focus prevention strategies and the control of human infections. In the specific case of WNV, a major aspect is the role of the different potential host species. Within this review of the literature, we aimed to summarise the existing knowledge on host selection among WNV-transmitting *Culex* mosquitoes. Our research found and reported nine studies that considered forage ratio as index of host selection [22,33–40].

In the analysis of the proportion of blood meals taken, American Robin (*Turdus migratorius*), House Sparrow (*Passer domesticus*), and House Finch (*Haemorhous mexicanus*)—all passerine species—were the most common blood meals, throughout the different studies. These results are comparable to previous findings [27,42,43]. Molaei and colleagues [42] reported that 38% and 10% of blood meals taken by *Cx*. *pipiens* derived from American Robin and House Sparrow, respectively. Similarly, 37% of blood meals taken by *Cx*. *restuans* derived from American Robin [42]. Moreover, Savage and colleagues [43] found that throughout different species of *Culex* mosquitoes (*Cx*. *pipiens*, *Cx*. *restuans*, *Cx*. *erraticus*, and *Cx*. *quinquefasciatus*), the most common blood meals were derived from American Robin, Common Grackle, and Northern Cardinal. Molaei and colleagues [27] reported that the majority of blood meals taken by *Cx*. *quinquefasciatus* derived from Columbiformes (Mourning Dove, White-winged Dove) and Passeriformes birds (House Sparrow, House Finch, Gray Catbird, and American Robin). In the specific case of *Cx*. *pipiens*, the predilection for American Robin was also proved in experimental settings [44]. *Cx*. *pipiens* mosquitoes significantly chose American Robin over European Starling and House Sparrow, also after accounting for weight, age, and sex of the animal and environmental parameters [44]. This distribution of favourite blood meals might have been expected when considering that the majority of the studies were conducted in the US. WNV first entered the Americas in 1999 [45]. The first outbreak is considered to be the result of the amplification effect of House Sparrows, while different species such as American Robin gained a major role as WNV spread in the Continent [18]. These avian species are considered to have had a paramount role in the transmission of WNV in the Americas, due to their ability to develop higher levels of WNV viremia for longer times, compared to other avian species. Komar and colleagues [20] reported both species were infectious in average for 4.5 days. The level of viremia in American Robin ranged between 5.8 plaque-forming units [PFUs]/ml of serum on the first day since the infection, 8.9 PFU/ml on the second, and 7.3 PFU/ml of the third. This higher viremia allowed—in turn—for the development of a principal vector role for *Cx*. *pipiens* and *Cx*. *quinquefasciatus*, which need higher titres to become infected, compared to other *Culex* mosquitoes [18,20]. Similarly, the level of viremia in House Sparrow ranged between 7.8 PFU/ml on the first day since the infection to 10.3 PFU/ml on the fourth day. However, following Del Amo and colleagues [46], House Sparrow present with higher host competency for North American WNV strains (NY99) compared to Southern European strains. The specific evolution of WNV in North America can be also seen in our results when considering the studies that were conducted in Meso- or South America. The relative abundance of blood meals in the studies by Mendenhall [34] and by Estrada-Franco and colleagues [38], conducted in Colombia and Mexico, respectively, included different species. This could be explained considering that the feeding patterns of *Culex* mosquitoes differ based on the host availability in the specific setting, which might change drastically when moving relative small distances [23,47]. This aspect is present also in the result of the study by Thiemann and colleagues [39], which was conducted in the vicinity of a breeding site for herons. In this study, the larger proportions of blood meals derived from herons. However, before heron’s breeding season, passerine birds (especially American Robin and Yellow-billed Magpie) and Columbiformes (Mourning Dove) were significantly preferred by *Cx*. *tarsalis*. Besides potential bias in the study (e.g., nonrandom selection of mosquitoes collection sites, which would hinder the effect of common roosting), this shift in feeding patterns during herons’ breeding season might then be influenced by the presence of herons’ nestlings. Nestlings—when not sheltered by parents—are easier to fed on for mosquitoes, due to their lower mobility and plumage. This higher sensitivity to mosquito bites extends also to the parents, especially in species that spend long time in the nest without moving. Studies that observed changings in feeding patterns over time reported higher proportion of blood meals taken from several species such as Cooper’s Hawk (*Accipiter cooperii*) and American Crow during their nesting period [47,48]. This aspect was highlighted in the work by Egizi and colleagues [48], who speculated that the shift in mosquitoes’ feeding patterns might be the result not of avian species being less available (e.g., due to migration) but rather of avian species being more available (e.g., due to their nesting behaviour) during nesting season. This speculation seems to be in line with what was observed in the study by Thiemann and colleagues [39], which reported a preference for American Robin, Yellow-billed Magpie, and Mourning Dove from *Cx*. *tarsalis* during the early season, before the herons’ breeding period. The finding of the preference for American Robin in the early season was confirmed also by Kent and colleagues [49], by Kilpatrick and colleagues [50], and by Molaei and colleagues [42]. The latter study reported a decreasing trend of the proportion of blood meals taken on American Robin from June to October. Conversely, a different trend for blood meals taken on American Robin was reported by Montgomery and colleagues [31] for *Cx*. *pipiens* complex. In this study, no blood meals were detectable in May, while June, July, and August presented with constantly growing proportion of blood meals taken on American Robin. Nevertheless, the subsequent shift in feeding patterns after herons’ breeding season towards mammalian-derived blood meals reported by Thiemann and colleagues [39] could generate speculation of a combined effect of higher availability (e.g., breeding and nesting behaviour) and lower availability (e.g., migration) in shaping feeding patterns.

More generally, our results support the presence of host selection towards Passeriformes birds. American Robin, Northern Cardinal (*Cardinal cardinalis*), House Finch, Northern Mockingbird (*Mimus polyglottos*), Brown Trasher (*Toxostoma rufum*), Magpie (*Pica pica*), and Yellow-billed Magpie (*Pica nuttalli*) were all significantly preferred by at least one species of mosquitoes. Similarly, House Sparrow and Common Grackle (*Quiscula quiscula*) were significantly preferred from certain mosquito species and avoided from others. Northern Cardinal was already reported as common blood meal for *Cx*. *pipiens* and *Cx*. *restuans* by Patrican and colleagues [51]. Moreover, similar to other studies, no significant preference but rather an avoidance was found throughout the studies included in this review for American Crows (*Corvus brachyrhynchos*). Due to their high mortality rates during the first WNV outbreaks in North America, corvids, in general, and American Crows, in particular, have been often credited a major role in WNV dynamics [20]. Corvids showed the potential to act as competent hosts for WNV, in experimental studies [20,52,53]. Komar and colleagues [20] reported that—among other birds experimentally infected with the North American WNV strain (NY99)—American Crows, Fish Crows (*Corvus ossifragus*), and Black-billed Magpies (*Pica hudsonia*) presented with a viremia lasting between 3.8 and 5 days, with level of viremia ranging between 5.8 and 10.2, 1.3 and 8.9, and 4.0 and 8.8 PFU/ml of serum, respectively. Moreover, both Carrion Crows (*Corvus corone*) [53] and Magpies [52] were reported to be competent hosts for WNV lineages 1 and 2. However, in blood meal analysis, corvids, in general, and American Crows, in particular, are often underrepresented [27,42,51]. A possible explanation was provided by Wheeler and colleagues [47], wherein the authors reported that American Crows might play an important role as early-amplifying hosts for WNV, because of their nesting season and characteristics, as well as high viremia titres. Another specific aspect for which American Crows were considered important in WNV dynamics is the potential bird-to-bird transmission in communal roosting sites [54]. However, the frequency with which they are fed upon from *Culex* mosquitoes was significantly associated with the proximity of the nest to the mosquitoes’ capture site [47]. This could explain the results by Kilpatrick and colleagues [36] on Fish Crows. They reported Fish Crows to be fed upon by *Cx*. *pipiens* and *Cx*. *restuans* more than what would be expected if feeding was based on chance alone (25 and 11 times as much, respectively, at 2 different sites). However, the authors questioned their effective impact on WNV transmission dynamics, based on their rarities at each site. They concluded that Fish Crows were responsible for 2% of infected mosquitoes, compared to, e.g., 59% of mosquitoes infected by American Robin [36]. The role of Crows and American Crows—in particular—and of bird-to-bird transmission—in general—presents therefore still with a lack of clarity and could benefit from further research.

In all the included studies, WNV-transmitting *Culex* mosquitoes, although to various extent ornithophilic, presented with opportunistic feeding behaviour. The proportion of avian- and mammal-derived blood meals in the included studies ranged from approximately 100% avian (*Cx*. *restuans*, *Cx*. *quinquefasciatus*, *Cx*. *pipiens* according to Kothera and colleagues [37]; *Cx*. *quinquefasciatus* according to Hannon and colleagues [33]; and *Cx*. *tarsalis* in early season according to Thiemann and colleagues [39]) to 30% to 40% of mammal-derived blood meals (*Cx*. *quinquefasciatus* according to Mackay and colleagues [40]; and *Cx*. *erraticus* according to Mendenhall and colleagues [34]). Previous studies on the topic reported *Cx*. *pipiens* and *Cx*. *restuans* to be largely ornithophilic, *Cx*. *erraticus* largely mammalophilic, and *Cx*. *quinquefasciatus* and *Cx*. *tarsalis* to exhibit the broadest opportunistic behaviour [28,42,43,55,56]. More in detail, Molaei and colleagues [42] reported 93%, 2%, and 4% of blood meals taken by *Cx*. *pipiens* to be avian-, mammal-derived, and mixed, respectively, while 100% of blood meals taken by *Cx*. *restuans* were avian-derived. Apperson and colleagues [55] reported a ratio 23:1 for blood meals taken on birds and on humans, respectively, by *Cx*. *pipiens*. Similarly, Campbell and colleagues [56] reported *Cx*. *pipiens* feeding on 17 avian species and 1 mammal species (humans). Thiemann and colleagues [28] reported at different sites a proportion of single-source blood meals taken on mammals that ranged between 0% and 9%, while the corresponding proportion of blood meals taken on birds ranged between 91% and 98%. Savage and colleagues [43] observed a slightly more opportunistic feeding behaviour from *Cx*. *pipiens*. The authors reported 73%, 14%, and 4% of blood meals having avian, mammal, or mixed origin, respectively [43]. In our results, this tendency of *Cx*. *pipiens* to feed largely on birds was reported by Kothera and colleagues [37]. In this study, 3 blood meals out of 840 taken by *Cx*. *pipiens* were from mammals (2 from humans). Conversely, the study from Hamer and colleagues [22] reported a significant percentage of blood meals taken on mammals and predominantly humans. A potential explanation for this discrepancy is the absence of control for the genetic ancestry of the form of the observed *Cx*. *pipiens* (*Cx*. *pipiens* form pipiens, *Cx*. *pipiens* form molestus, or hybrids). According to Kilpatrick and colleagues [57], the probability of blood meals taken on mammals (including humans) was proportional to the fraction of genetic ancestry with *Cx*. *pipiens* from molestus. This difference in host preferences between *Cx*. *pipiens* form pipiens (more ornithophillic) and *Cx*. *pipiens* form molestus (more mammalophilic) was observed also in experimental setting in the work by Fritz and colleagues [58].

*Cx*. *quinquefasciatus* presented the widest feeding spectrum among the observed mosquitoes, with mammal-derived blood meals ranging from 2% according to Hannon and colleagues [33] to 39% according to Mackay and colleagues [40]. This result might seem to disagree with previous works. Zinser and colleagues [59] reported a 50% and 32% of blood meals to be human- and bird-derived, respectively. Similarly, Molaei and colleagues [27] reported 39% and 52% of blood meals to be bird- and mammal-derived, respectively. However, in this study, only 3 human blood meals were collected. A potential explanation for this difference in results might be in the heterogeneous host availability in the different studies. This was also suggested in the study by Zinser and colleagues [59], who contextualised the observed results in light of an elevated variability in host utilisation among *Cx*. *quinquefasciatus*.

In the study by Kothera and colleagues [37], *Cx*. *restuans* blood meals were exclusively from avian species. Conversely, in the work by Hamer and colleagues [22], *Cx*. *restuans* exhibited a tendency to ornithophilic feeding behaviour (80% and 15% of blood meals were avian- and mammal-derived, respectively). Previous works on *Cx*. *restuans* agreed on this high variability of blood meals composition [43,48]. Egizi and colleagues [48] reported that early-season blood meals from *Cx*. *restuans* were almost entirely avian-derived (although human-derived blood meals were present). Conversely, Savage and colleagues [43] reported 62% of *Cx*. *restuans* blood meals to be avian-derived (most commonly American Robin, Common Grackle, and Northern Cardinal).

In the study by Thiemann and colleagues [39], *Cx*. *tarsalis* presented with exclusively avian-derived blood meals in the early season, which shifted to 12% of mammal-derived blood meals in the late summer season. A similar shift in blood-meals composition was reported also from Kent and colleagues [49]. The authors considered *Cx*. *tarsalis* to feed on both avian (especially Mourning Dove and American Robin) and mammals (especially Domestic Cow). Human blood tended to be more present in late summer [49]. Similarly, Molaei and colleagues [30] reported that *Cx*. *tarsalis* fed significantly more often on Mourning Dove and House Finch. Campbell and colleagues [56] reported that *Cx*. *tarsalis* fed on 30 avian and 11 mammal species (most commonly American Robin, Domestic Cow, and Yellow-billed Magpie). A preference for Mourning Dove and Yellow-billed Magpie is present also in the included study by Thiemann and colleagues [39]. However, both Thiemann and colleagues [28] and Campbell and colleagues [56] agreed that feeding patterns of *Cx*. *tarsalis* are highly different based on hosts availability. Mammal-derived blood meals ranged from 3% to 29% in the different sampling sites [28].

As previously mentioned, all observed mosquitoes fed on humans as well. Thus, our results suggest that all observed mosquitoes could have the potential to transmit to humans zoonotic pathogens, even if to different degrees. For some mosquitoes, this aspect has been already documented. Molaei and colleagues [27] reported that blood meal patterns from *Cx*. *quinquefasciatus* were compatible with human cases in Harris County, TX. Similarly, Molaei and colleagues [30] considered *Cx*. *quinquefasciatus* the primary WNV vector in Southern California. For other mosquitoes, even if percentage of blood meals taken on humans might be negligible, the fact that their opportunistic feeding behaviour includes human blood meals retains some level of potential to act as a vector for zoonotic pathogens. Conversely, Thiemann and colleagues [28] discussed that few blood meals from *Cx*. *pipiens* complex and *Cx*. *tarsalis* analysed in the metropolitan area of Los Angeles, CA, were human derived. The authors, therefore, considered the two mosquito species to have a marginal role as bridge vector, as the study area recorded several WNV outbreaks. Nevertheless, considering the density of the population in the aforementioned areas, even such a marginal vector role could lead to significant amounts of cases each season.

### Implications for further research

All but one, which was conducted in Italy, of the included studies took place in the Americas. As observable in our results and other works on the topic, mosquitoes’ host selection can be largely influenced by the immediate environment and hosts availability [47,60]. In addition to this, several studies have observed that Palearctic and Nearctic birds present with different sensitivity to WNV, especially in terms of clinical symptoms and mortality [61,62]. It is still unclear whether this aspect can be explained by differences in the viral strains, coevolution with WVN or cross-immunization with different Flavivirus of Palearctic birds, or different vector ability of the mosquitoes [61]. However, for these reasons, together with the different avian population in the European regions, the bulk of the results of the study summarised in this review cannot be directly extended to other settings. In recent years, WNV has been severely present in Southern Europe [8]. However, even if studies on potential host competence of common avian species (e.g., Red-Legged Partridge [*Alectoris rufa*]) [63] as well as on host selection in Europe and United Kingdom are present [64–66], the ones considering forage ratio are rare. Hence, such a study based in the different European regions (e.g., other than Italy, for which a study already exists; [35]) could help to further disentangle the relation between mosquitoes and Palearctic birds. In addition, another study exploring the role and the host competence in different European Regions of avian host, which were observed to be preferred (e.g., Common Blackbird, Magpie) or avoided (e.g., European Sterling, Rock Pidgeon), should be considered.

### Implications for clinicians and policy makers

Our results suggest that even if within a host selection strategy, feeding patterns of *Culex* mosquitoes vary based on the environment and the host availability. In a biodiverse environment, in which mosquitoes are presented with high density of potential hosts, a so-called “dilution effect hypothesis” (DEH) might occur [67]. DEH is based on the presence of incompetent reservoir hosts (e.g., non-passerine birds). The WNV inoculated after a blood meal on these incompetent hosts would not generate sufficient viremia to reinfect mosquitoes, de facto not taking part in the amplification cycle. However, to date, controversial results are present on whether a richer biodiverse environment could result in a protective effect on the transmission of arthropod-borne diseases or the opposite [67–70].

As we observed, mosquito feeding patterns are influenced from host availability. Hence, the presence of several incompetent hosts might indeed lead to blood meals being taken out of the amplification cycle. In the specific case of WNV, this speculation has been studied by Ezenwa and colleagues [71], who observed a negative association between the density of non-passerine bird species and the WNV infection prevalence as well as density of infected *Culex* mosquitoes. Thus, further studies on the specific applicability of DEH on WNV for policy making could be considered.

### Limitations

When interpreting the results of this analysis, it is important to consider also its limitations. The included studies considered different *Culex* species, with different distributions and habitats, both in terms of geographical and climatic areas they are present in, as well as whether they prefer living in urbanized, rural, or semirural environments. These aspects have been observed to deeply influence the composition, diversity, and abundance of potential hosts the mosquitoes can feed upon [60]. Similarly, it was not possible to assess the role of aggregates of vertebrate hosts (e.g., communal roosting sites) to attract WNV-transmitting *Culex* mosquitoes. For these reasons, the results should not focus on the single host species per se, but rather on the characteristics of the hosts selected in the different settings. Moreover, the studies considered different strategies to evaluate the population of possible hosts in the selected environment: The majority of studies considered point surveys at the considered location, while Mackay and colleagues [40] used the North American Breeding Bird Atlas and Hannon and colleagues [33] used online repository of birdwatching checklists. This heterogeneity in evaluating the relative abundance of potential hosts might lead to noncomparable results. Nevertheless, this limitation has been already reported in studies that considered forage index [23].

As previously mentioned, these results might not be directly translated to other settings than the ones in which were developed. In addition, even if this review was conducted with a clear methodological structure, only one reviewer extracted the included studies and their information, rendering the review not systematic and therefore—by definition—more prone to reporting bias. However, considering the small number of studies retrieved and their intrinsic differences such as different countries, vector, and host availability, we considered that a more systematic approach to this review would not have allowed this study to draw stronger conclusions.

Finally, when interpreting the results of this review, it is important to consider that the preference or avoidance of a certain host species does not immediately translate to a major role in the WNV transmission dynamics. This is because, besides the role as a potentially favourite host, there must be also the host competency of the single species. For this reason, the capacity of each species to reinfect mosquitoes, both in terms of sufficient level of viremia as well as availability of the host, should be observed in detail also with specific studies. This approach could help disentangling the role of the different hosts in WNV transmission dynamics and, thus, to focus preventive strategies.

## Conclusions

We aimed to summarise existing knowledge on host selection in WNV-transmitting *Culex* mosquitoes. Our results suggest that all observed mosquitoes present opportunistic feeding behaviours, having taken blood meals both of avian and mammal species. In addition, the constant presence of human blood meals suggests that all the observed mosquitoes have the potential to act as bridge vectors for WNV infection to humans. WNV-transmitting *Culex* mosquitoes presented in all the included studies a pattern of preference and avoidance. This result suggests that they do not take blood meals solely based on the hosts’ availability, but still operate a certain level of selection, independently from the different settings.

Whereas some level of generalization can be inferred from the included articles, they present with sensitive differences, among which the species of mosquito observed, the study settings (e.g., geographical area, population of hosts) and design (e.g., strategy to collect mosquitoes and evaluate abundance of hosts, time frame of the vector collection). These differences disallow for generalization of the results in settings others than the ones of each study and suggest caution when interpreting them.

## Related manuscripts

NR, AF, FF, JGR, and NS declared no related manuscript in consideration elsewhere.

## Disclaimer

The views expressed in this article are purely those of the authors and may not, under any circumstances, be regarded as an official position of the European Commission.

**Key Learning Points**

- All observed West Nile virus (WNV)-transmitting *Culex* mosquitoes present with opportunistic feeding behaviours.
- All observed WNV-transmitting *Culex* mosquitoes can potentially have a role in bridging the infection to humans.
- All observed WNV-transmitting *Culex* mosquitoes present patterns of selection and avoidance of potential hosts

**Top Five Papers**

1. Boreham PF, Garrett-Jones C. Prevalence of mixed blood meals and double feeding in a malaria vector (*Anopheles sacharovi* Favre). Bull World Health Organ. 1973;48(5): 605–14.
2. Lardeux F, Loayza P, Bouchité B, Chavez T. Host choice and human blood index of *Anopheles pseudopunctipennis* in a village of the Andean valleys of Bolivia. Malar J. 2007;6(1):8.
3. Chaves LF, Harrington L, Keogh C, Nguyen A, Kitron U. Blood feeding patterns of mosquitoes: random or structured? Front Zool. 2010;7(1):3.
4. Mann J, Washington M, Guynup T, Tarrand C, Dewey E, Fredregill C, et al. Feeding Habits of Vector Mosquitoes in Harris County, TX, 2018. J Med Entomol. 2020;57(6):1920–29.
5. Molaei G, Andreadis T, Armstrong P, Bueno Jr R, Dennett J, Real S, et al. Host feeding pattern of *Culex quinquefasciatus* (Diptera: Culicidae) and its role in transmission of West Nile virus in Harris County, Texas. Am J Trop Med Hyg. 2007;77(1):73–81.

## Advantages

1. The results of this review provide a summary of the existing knowledge and gaps on host selection and forage ratio in West Nile virus (WNV)-transmitting *Culex* mosquitoes.
2. These results could foster further research on the role of the different potential host species in the transmission cycle of WNV.
3. In addition, these results could support the development of research projects and public health interventions to disentangle and limit the transmission cycle of WNV at the local level.

## Disadvantages

1. Generalization of the results of this review is challenging because of observed local difference in host selection patterns.
2. Similarly, the different studies used different techniques to define the population of potential hosts (point count surveys, citizen science data, etc.), and for this reason, the comparison of the results between different studies should be conducted with care.
3. In addition, the preference or avoidance of a potential host species does not necessarily translate in implications for WNV transmission cycle.
